# Electrospun textiles from decellularized bovine pericardium and polyvinyl alcohol (PVA) supporting blood coagulation: innovative approach in biomaterials for research purposes

**DOI:** 10.3389/fbioe.2025.1539759

**Published:** 2025-08-11

**Authors:** Luca Di Nunno, Veronica Pagani, Elena Canciani, Gerardina Ruocco, Mattia Spedicati, Maria Talmon, Luca Fusaro, Dalila Di Francesco, Simona Casarella, Manuela Rizzi, Francesca Boccafoschi

**Affiliations:** ^1^ Laboratory of Human Anatomy, Department of Health Sciences, University of Eastern Piedmont, Novara, Italy; ^2^ Laboratory for Biomaterials and Bioengineering, Department of Min-Met-Materials Engineering, University Hospital Research Center, Regenerative Medicine, Laval University, Laval, QC, Canada; ^3^ Department of Mechanical and Aerospace Engineering, Politecnico di Torino, Torino, Italy; ^4^ Interuniversity Center for the Promotion of the 3Rs Principles in Teaching and Research, Pisa, Italy; ^5^ Laboratory of Pharmacology, Department of Pharmaceutical Sciences, University of Eastern Piedmont, Novara, Italy

**Keywords:** nanofibers, electrospinning, decellularized extracellular matrix, thromboelastographic analysis, regenerative medicine, blood clotting

## Abstract

**Introduction:**

Current hemostatic agents face several limitations, including reduced effectiveness in controlling massive bleeding or preventing thrombogenic events. Functional bleeding control could allow time for further treatment and decrease mortality rates. Using suitable hemostatic agents may improve surgical outcomes by eliminating avoidable complications. Among all the patches available on the market, nanofibrous materials offer several advantages, among which the possibility to be properly designed in order to meet specific requirements related to bioactivity and biodegradability. Different patch formulations to support blood clotting are characterized in the present study.

**Methods:**

The approach used is based on electrospun decellularized bovine pericardium (dECM) blended or layered with polyvinyl alcohol (PVA) and crosslinked by (3‐Glycidyloxypropyl)trimethoxysilane (GPTMS). Scanning electron microscopy and energy-dispersive X‐ray spectroscopy were used in order to characterize the scaffold’s morphology and chemical composition. Mechanical properties were evaluated using a tensile stress test, while wettability was measured using contact angle analysis. The electrospun textiles’ surface interaction with whole blood and platelet rich plasma (PRP) was also examined. Thromboelastography (TEG) and in vitro clotting assays were performed in order to evaluate the clot formation, while flow cytometry was used to verify platelet activation. Finally, in order to evaluate the biological response to the degradation by‐products of the electrospun textiles, cell viability was characterized through an indirect toxicity test using primary normal human dermal fibroblast (NHDF) as experimental model.

**Results and Discussion:**

Overall, the nanofibers-based textiles, optimized through blending and layering of dECM and PVA, successfully support stable clots. These biomaterials represent a valuable starting point for future research aimed to nanofibers’functionalization according to the desired application.

## 1 Introduction

Hemorrhage is one of the major causes of trauma-related fatalities, accounting for 30%–40% of death, especially in emergencies. A significant percentage of these fatalities (33%–56%) occurs during the prehospital phase, and even patients who receive prompt care may face with premature death due to uncontrolled bleeding and coagulation issues. In a context where over 300 million surgical procedures are performed annually, with a high incidence of preventable complications, effective bleeding control remains a critical worldwide clinical challenge (Lewis et al., 2018).

Conventional methods for controlling bleeding are often inadequate, particularly in the case of severe trauma or complex surgical procedures, where current hemostatic technologies reveal considerable limitations. This urgent need for improved tools in more effective bleeding management, particularly in the early post-traumatic or surgical phases, has stimulated a growing interest in advanced biomaterials research (Du et al., 2023). Modern wound dressings are designed to accomplish multiple essential functions, such as reducing bleeding, accelerating coagulation, protecting against infection, absorbing exudate, preventing maceration, and promoting tissue regeneration (Zagho et al., 2018).

Among the alternatives to conventional methods, hemostatic nanofiber patches offer an innovative approach designed to adhere effectively to wounds, promote coagulation, and accelerate healing. With their porous structure and ability to integrate bioactive substances, these patches offer significant advantages over traditional bandages or gauze. Furthermore, by leveraging nanotechnology, these advanced solutions provide a promising approach to hemostasis management, potentially reducing hemorrhagic complications and improving patient outcomes (Du et al., 2023).

Tissue engineering has introduced new possibilities with electrospun materials, providing a highly versatile platform for developing hemostatic patches. Electrospinning is an efficient method for producing nano-scale fibrous structures with a large surface-area-to-volume ratio and enhanced water retention properties, making these materials suitable for blood-contact applications (Wu et al., 2022). The most promising strategies involve mixing or stratifying different polymers to obtain optimal properties for specific applications (Socci et al., 2023). In this study, new nanofiber-based textiles, obtained by combining synthetic and natural polymers, has been characterized, with particular attention to the ability of supporting hemostasis and, thus, driving tissue regeneration, offering extraordinary potential for improving clinical outcomes (Canciani et al., 2021).

The combination of decellularized extracellular matrices (dECM) based on bovine pericardium and polyvinyl alcohol (PVA) may represent an innovative approach in developing electrospun textile to guide bleeding control, and support cellular responses.

PVA is widely used as a scaffold material in tissue engineering because of its high solubility in water, non-toxicity, and oral safety, allowing easy miscibility at 37°C with the dECM-based solutions. Moreover, in combination with dECM, PVA contributes to enhance the overall mechanical tensile properties of the system (Santos et al., 2014).

dECM demonstrates excellent biological performance compared with other commonly used natural materials, such as collagen. dECM-based hydrogels contain high concentrations of extracellular matrix (ECM) proteins (collagen, fibronectin and laminin) and could simulate subcutaneous ECM components and actively participate in the hemostatic process, also because they closely mimic the native ECM (Brown et al., 2022; Cai and Weng, 2023; Di Francesco et al., 2024). dECM from bovine pericardium retains a rich, native ECM composition, including the newly discovered bioactive matrix-bound nanovesicles inside (Di Francesco et al., 2024). The richness and complexity of bovine pericardium dECM has shown extraordinary regenerative potential, thanks to its ability to induce potent cell signaling and to modulate the immune system, emphasizing its role in providing structural support and delivering biochemical signals fundamental to the regeneration process (Giobbe et al., 2019; Di Francesco et al., 2022; Di Francesco et al., 2024). These environmental signals are not limited to ECM tissue-specific proteins but also include soluble factors absorbed within the ECM protein network (Giobbe et al., 2019). Literature increasingly supports the application of dECM in biomedical applications, particularly in tissue regeneration (Yu and Zhong, 2021).

This study proposes to optimize the electrospun materials involving bovine pericardium dECM/PVA textiles to support blood clotting. The morphological and mechanical properties, wettability, and hemostatic properties of electrospun hybrid matrices derived from decellularized bovine pericardium have been evaluated.

## 2 Materials and methods

### 2.1 Matrix from decellularized bovine pericardium

The decellularized bovine pericardium matrix (dECM) has been provided by Tissuegraft srl. (Italy). Briefly, bovine pericardium, sourced from by-products of the food industry, was processed to eliminate cellular components and nucleic acids. Freeze-dried pericardium dECM was enzymatically digested to obtain a hydrogel intended for research and medical use (Italian patent number 102020000007567, patented on 29 April 2022; International patent number PCT/IB 2021/052779 submitted on 2 April 2021) (Di Francesco et al., 2022; Di Francesco et al., 2024).

### 2.2 Electrospinning process

Hemostatic patches were fabricated by electrospinning using dECM bovine pericardium-based solution, gelatin type A, and polyvinyl alcohol (PVA) in a solvent system of water and glacial acetic acid (all the reagents were provided from Sigma Aldrich, Italy). Patches were fabricated in either blend or layer form. The total volume of electrospun solution was kept constant at 10 mL for each sample.

The electrospinning solutions were prepared by dissolving the different polymers in water and acetic acid 1:1 v/v. Briefly, as controls, PVA and gelatin was dissolved at 50°C under constant stirring for 30 min, while dECM was created dissolving 10% w/v gelatin at 50°C, let cooling until reaching 37°C before adding 10% w/v dECM with the crosslinker GPTMS (3-Glycidoxypropyl-) trimethoxysilane (0.200 μL/g gelatin). Layer 10 was obtained with a sequential electrospinning approach. Electrospinning procedure was performed by first using the PVA solution following by dECM solution. Blend 10 was obtained by dissolving PVA and gelatin by heating at 50°C for 30 min. The solution has been used when 37°C were reached and mixed with 10% dECM lyophilized powder. Once the final solution was homogeneous, the crosslinker was added.

The parameters used during electrospinning procedure are reported in detail in Table 1. During the electrospinning process the relative humidity was maintained at 20%, and the process temperature was set at 30°C. The collector was set at a fixed rotation speed of 750 rpm, with a needle-to-collector distance of 10 cm, a maximum offset of 60 mm, and a linear speed of 20 mm/s. After the electrospinning process, all the samples were dried under air flow at room temperature (RT) overnight. Sterilization procedure has been performed using the UV-C lamp (∼254 nm) provided in the class II biological safety cabinet (Angelantoni Life Science, Italy - Steril VHB 48 C2 biological cabinet). Sheets were sterilized by exposing each side to the UV light for 30 min, maintaining a distance of 30 cm from the light source. UV-C irradiation intensity in the system was approximately 0.12–0.15 mW/cm^2^.

**TABLE 1:** Electrospun samples formulation: polymers concentration expressed in % w/v and process parameters.

SAMPLES	PVA	Gelatin	dECM	Flow mL/h	Electric field KV	Distance Cm	Needle Ga
PVA	10%	0%	0%	1	15	10	20
10% dECM	0%	10%	10%	1.5	35	10	20
Layer 10	10%	10%	10%	1 and 1.5	15 and 35	10 and 11	20 and 24
Blend 10	5%	5%	10%	1	35	11	20

### 2.3 Surface analyses: SEM and EDS analysis

The electrospun patches were morphologically and chemically characterized with a scanning electron microscope (SEM) JSM-IT500A/LA in TouchScope™ equipped with an energy-dispersive X-ray spectroscopy (EDS) camera (JEOL, Japan). The electrospun fibers were mounted on the stub using carbon tape and sputter-coated with gold (2 min). Images were acquired at 2500x, 5000x, 10000×, and 20000× magnification to visualize surface morphology. The average fiber diameter and pore size were calculated from SEM images using ImageJ (NIH) and Mountains10 (Digital Surf) software. The material/pores interface was detected by thresholding the intensity profile, using the software’s ‘material ratio’ function (59.55%) as a segmentation criterion. For 3 samples, 3 random areas were analyzed at the magnification of 5000x, and data was processed using Excel and plotted in GraphPad (Prism9).

### 2.4 Mechanical characterization

To investigate the deformability of electrospun membranes, uniaxial tensile tests were performed at room temperature in dry conditions using the MTS QTest/10 (MTS Systems S.r.l., Torino, Italy, equipped with Testwork 4 software (MTS Systems S.r.l., Torino, Italy). A load of 10 N was applied, and a consistent testing speed of 1 mm/min was maintained throughout all the measurements. Six samples were obtained from 3 different nanofiber sheets and punched into standardized dog-bone specimen (Width: 5 mm, Gauge length: 15 mm), and their thickness was measured with a digital micrometer to check batch-to-batch variability.

The specimen were securely fixed on custom-made grips preloaded slightly before applying the strain deformation. Stress (σ) was calculated by dividing the applied load value (N) by the initial cross-sectional area (mm^2^), while strain (ε) was defined as the change in length relative to the original length. The resulting stress-strain (σ-ε) curves displayed distinct elastic, yield, plastic, and failure points. The elastic region exhibited a linear relationship between stress and strain, while the plastic region indicated permanent deformation. The tensile modulus was determined from the slope of the elastic region, and the maximum strain was calculated at the failure point.

### 2.5 Contact angle measurements

To evaluate the effect of the membrane composition on wettability, static contact angle measurements were performed at room temperature using Milli-Q water. Measurements were conducted in triplicate at three time points: immediately after droplet deposition, after 10 s, and after 30 s (Spedicati et al., 2022). Static contact angles were measured using a 2 µL drop of MilliQ water in triplicate at three-time points: immediately after deposition (time 0), at 10 s (time 1), and at 30 s (time 2). The average values of the static contact angles and the relative standard deviations were obtained and analyzed using the Drop Shape Analyzer equipped with Advanced software (KRÜSS GmbH—KRÜSS Scientific Instruments), using the sessile drop method.

### 2.6 Hemocompatibility characterization

Healthy donors human whole blood was provided by the Transfusion Service of the Ospedale Maggiore della Carità (Novara, Italy) after authorization from the local Ethics Committee (Comitato Etico Interaziendale Maggiore della Carità, Novara; authorization document 88/17). Eight healthy volunteer donors (5 females and 3 males), aged 21–45 years were enrolled in the study. Donors had not taken any medications for at least 14 days before drawing and had no history of bleeding disorders. As positive control, EVITHROM^®^ Thrombin was reconstituted in ultrapure water (ETHICON, Inc., United States). Thrombin was used at the final concentration of 500 IU/mL.

#### 2.6.1 Thromboelastographic analysis

Sodium citrate at 3.2% v/v was added to the whole blood. Within 2 h from the collection, 1 mL of blood was added to each well of a 24-well plate containing 1 cm^2^ electrospun samples and incubated at 37°C for 30 min, with plastic serving as control. After the incubation 340 μL mL of blood were activated in kaolin solution (Haemonetics, Italy), inverted five times, and added to each thromboelastographic with thromboelastography (TEG) cup, preceded by 20 µL of 0.2 M CaCl_2_ solution to start the coagulation process. The TEG parameters, including reaction time (R), clotting time (K), Angle and maximum amplitude (MA) were assessed using a TEG^®^ 5000 Thromboelastographic Hemostasis Analyzer System (Haemonetics, Italy) at 37°C for 1 h (Fusaro et al., 2020). The results were analyzed using Excel and GraphPad (Prism9).

#### 2.6.2 *In vitro* coagulation test

The hemoglobin-free method was adapted from Huang et al. (2003) and it was used to describe the relative stability of the clots on the surface of the different samples. Briefly, 20 μL of freshly human-collected blood (without anticoagulant) were dropped onto 0.16 cm^2^ of each surface in a 48-well plate. After 0, 5, 10, 15, 30, and 45 min 500 μL of MilliQ water were added to halt the clotting process. After an incubation of 5 min, free hemoglobin was evaluated by transferring 100 μL of the resulting solution into a 96-well plate. Absorbance was measured at 540 nm using the Victor4X Multilabel Plate Reader (Perkin Elmer, Milan, Italy). The absorbance was used to colorimetrically measure the free hemoglobin released by red blood cells. In fact, cells not entrapped in a thrombus-like structure were hemolyzed resulting in a proportional absorbance increase. The test was performed in triplicate. Data were exported and mean/standard deviation was expressed as percentages compared to the plastic control. Relative stability of the clots on the surface was defined as the point at which 50% hemoglobin was released. The data was processed using Excel and GraphPad (Prism9).

#### 2.6.3 Clot morphometrical analyses: SEM microscopy

Additionally, 0.16 cm^2^ samples that were in contact with 20 μL blood and platelets for 30 min at 37°C were analyzed to qualitatively and quantitatively evaluate blood-surface contact. Whole blood was obtained from 3 healthy donors as described in section 2.7.1. Sample preparation included washing twice with cacodylate buffer 0.2 M, followed by fixation with 2.5% glutaraldehyde in 0.1 M Karnowski buffer for 24 h, and a final washing with 0.2 M cacodylate. Following, the samples were dehydrated by immersion in a series of ethanol solutions at increasing concentration (50%, 70%, 80%, 95%, and 100%) for 30 min each. Then, the samples were washed for 1 hour with hexamethyldisilazane (HMDS, VWR, Italy) to remove alcohol residues. Afterwards, the samples were soaked in HMDS overnight until the complete evaporation was obtained. All these steps were performed at room temperature working in a chemical hood assuring a vertical flow. Dried samples were mounted on the stub using carbon tape, sputter-coated with gold, and observed as previously described.

A morphometrical analysis of the clot was performed to support the hemocompatibility test adapting the methodology of Nalezinková et al. (2024). In brief, the analysis is based on the principle of statistical geometric probability, which states that the probability of a point falling on a specific structure is proportional to the relative area of that structure. This method allows to measure blood cells relative to the total surface. Morphometric analyses were performed using SEM images at 1000x magnification. A stereological grid of 280 test points was superimposed on each image to assess the relative stability of the clots on the surface of the different samples. Three SEM images for each patient were selected. The percentage of test points falling on clot components was counted, and a quantitative index of clot adhesion and persistence in contact with the samples was expressed as a percentage of the total area.

#### 2.6.4 Flow cytometry on platelets rich plasma

Blood was collected using 4.5 mL Vacutainer tubes containing 3.8% sodium citrate as anticoagulant (Becton Dickinson Vacutainer Systems, Plymouth, England). To obtain platelet rich plasma (PRP), the whole blood was transferred to a sterile tube and centrifuged at 200 *g* for 20 min at room temperature. At the end of the centrifugation step, the PRP fraction which appears just above the buffy coat, was carefully collected with a sterile pipette and transferred to a new sterile tube. HEP buffer (140 mM NaCl, 2.7 mM KCl, 3.8 mM HEPES, 5 mM EGTA, pH 7.4) was then added with a 1:1 ratio and then gently mixed by inversion. The resulting mixture was centrifugated at 100 *g* for 20 min at room temperature and the pellet was resuspended in plasma to obtain the PRP final fraction. Flow cytometry was used to assess the effect of the samples on platelet activation. Freshly isolated PRP was used to seed 500.000 platelets/cm^2^ per electrospun sample.

The activated PRP was incubated at 37°C for 5 min with the samples. An empty well and a well containing thrombin were used as negative and positive controls, respectively. The platelets were then fixed with 1% paraformaldehyde (PAF) for 15 min. The platelets were centrifuged at 800 *g* for 15–20 min at room temperature, and the supernatant was discarded. The pellet was resuspended in Tyrode’s buffer (134 mM NaCl, 12 mM NaHCO_3_, 2.9 mM KCl, 0.34 mM Na_2_HPO_4_, 1 mM MgCl_2_, 10 mM HEPES, pH 7.4) (Sigma, Italy), containing 15 mM Pyridoxal Phosphate Analog Compound (PPAC, Sigma, Italy) to prevent platelet aggregation. After activation, cells were immunostained with anti-CD62p-PE (Thermo-Fisher, Italy) and incubated for 1 h in the dark at 4°C. After incubation, the samples underwent three washes to remove excess antibodies. Fluorescence was quantified using Attune NxT (Life Technologies, Monza, Italy) instrument. Data was expressed as the percentage of positive and platelet activation was compared across different samples.

### 2.7 Cytocompatibility characterization

#### 2.7.1 Indirect cytotoxicity assay

The viability of normal dermal human fibroblasts (NDHF) in a medium conditioned by electrospun samples was evaluated.

6 cm^2^/mL of electrospun samples were placed in Dulbecco’s modified Eagle’s Medium (DMEM) supplemented with 10% fetal bovine serum (FBS), 2 mM Glutamine, 100 U/mL penicillin, and 100 μg/mL streptomycin (all from Euroclone, Italy), for up to 3 days. At 1 and 3 days, the media containing degradation by-products of the electrospun samples were collected for cytotoxicity assay (from now on referred to as conditioned medium).

Primary Normal Human Dermal Fibroblasts (NHDF-Ad CC-2511, Lonza, Switzerland) were cultured in DMEM supplemented with 10% FBS, 2 mM Glutamine, 100 U/mL penicillin, and 100 μg/mL streptomycin. Cells were cultured in 75 cm^2^ flasks (Falcon Corning, Germany) and maintained in an incubator set at a constant temperature of 37°C, with a relative humidity of 80% and 5% CO_2_. For all the experiments, cells were used up to passage 6.

NHDF were seeded at a concentration of 5000 cells/cm^2^ in 48 multiwell plates. After 1 day, the culture medium was replaced with the conditioned medium obtained from the electrospun samples after 1 and 3 days of degradation. At 1 and 3 days, 3-[4,5-dimethylthiazol-2-yl]-2,5 diphenyl tetrazolium bromide (MTT) assay was performed by adding 0.5 mg/mL of 3–4,5-dimethyl-2-thiazolyl)-2,5-diphenyltetrazolium bromide (Merck), diluted in DMEM without phenol red. After 4 h of incubation at 37°C, relative humidity of 80%, and 5% CO_2,_ the formazan crystals were solubilized by adding dimethyl sulfoxide (DMSO, Sigma-Aldrich). The absorbance of the solution was measured at a wavelength of 570 nm using a spectrophotometer (Victor x4, Perkin Elmer, Milan, Italy). The data were expressed as a percentage of relative viability normalized to control on day 1 and analyzed by Excel and GraphPad.

#### 2.7.2 Transwell migration assay

Transwell migration assay was performed by seeding 7500 cells/cm^2^ on the upper layer of a 0.3 μm transwell membrane (Merck in Milan, Italy) and placed in a conditioned medium. After 16 h, the transwell filter was removed and fixed with a 4% formaldehyde solution for 1 h and stained with a 0.1 mg/mL crystal violet solution (Sigma Aldrich, Milan, Italy) for 15 min. The transwells were then rinsed three times with water for 15 min, and the inner part of the membranes was then gently cleaned with a cotton swab. Five pictures were taken for each sample using an optical microscope, and the number of cells as well aspercentage area occupied by the migrated cells on the bottom side of the transwell membrane were analyzed.

The data were initially processed using an image color summarizer to quantify the areas occupied by the fibroblasts, and further processed using Excel and GraphPad.

### 2.8 Statistical analysis

All quantitative measurements are represented as means and standard deviation (SD). For the statistical analysis, tests based on blood were performed on at least 3 different donors. For the TEG analysis, eight donors have been included. For the *in vitro* coagulation test and flow cytometry assays, five donors have been evaluated. While Morphometric, mechanical, viability and migration tests have been repeated in triplicate in 3 independent experiments. Statistical analysis was performed using InStat3 software (Graphpad Instat Software Inc., United States), employing the Tukey-Kramer Multiple Comparisons Test. The significance level was set at α = 0.05 with *p-values* reported as follows: *p < 0.5, **p < 0.01, ***p < 0.001.

## 3 Results

**FIGURE 1:**
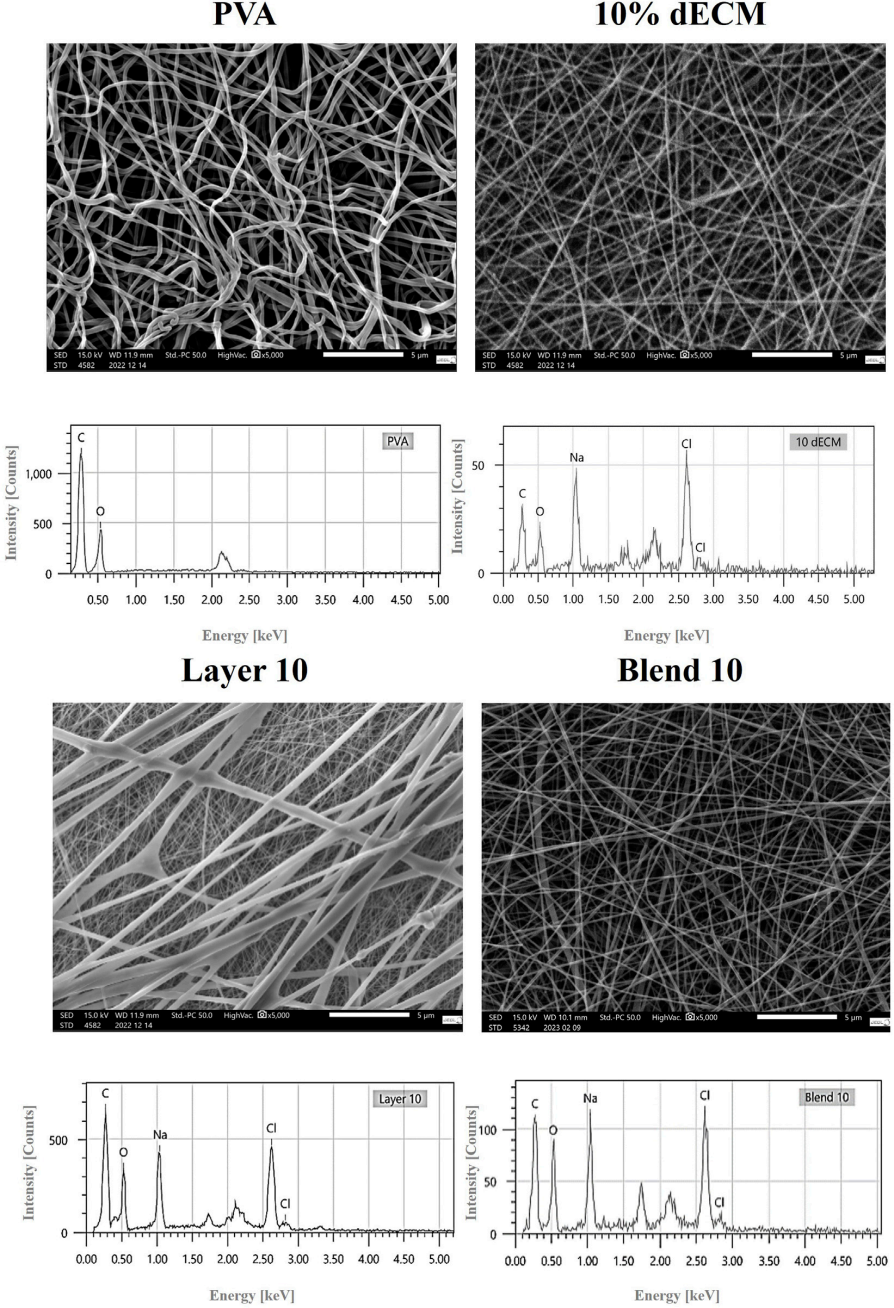
High-resolution SEM image (5000X magnification) provides detailed surface morphology. Relative EDS analyses are also shown.

All samples, including single polymer and hybrid PVA/dECM patches, were successfully obtained by means of electrospinning techniques employing layering and blending strategies. Figure 1 shows the microscopic appearance of the obtained fibers obtained by this methodological approach (Figure 1). In order to achieve a continuous and reproducible electrospinning process, different electrospinning parameters were optimized. The results showed easier processability of PVA with respect to gelatin and dECM samples. An optimal electric field of 15 kV was identified for PVA, while dECM required a higher voltage of 35 kV to be correctly accelerated toward the collector.

As expected, the physical properties of the solution, processing and environmental conditions influence the electrospinning process. These characteristics significantly impact the morphology of the fibers, allowing the customization of the process to meet specific requirements.

The EDS spectra analysis confirmed the presence of carbon and oxygen in all samples, while the samples containing dECM also showed the presence of sodium and chloride (Figure 1).

### 3.1 Morphometric analysis

**FIGURE 2:**
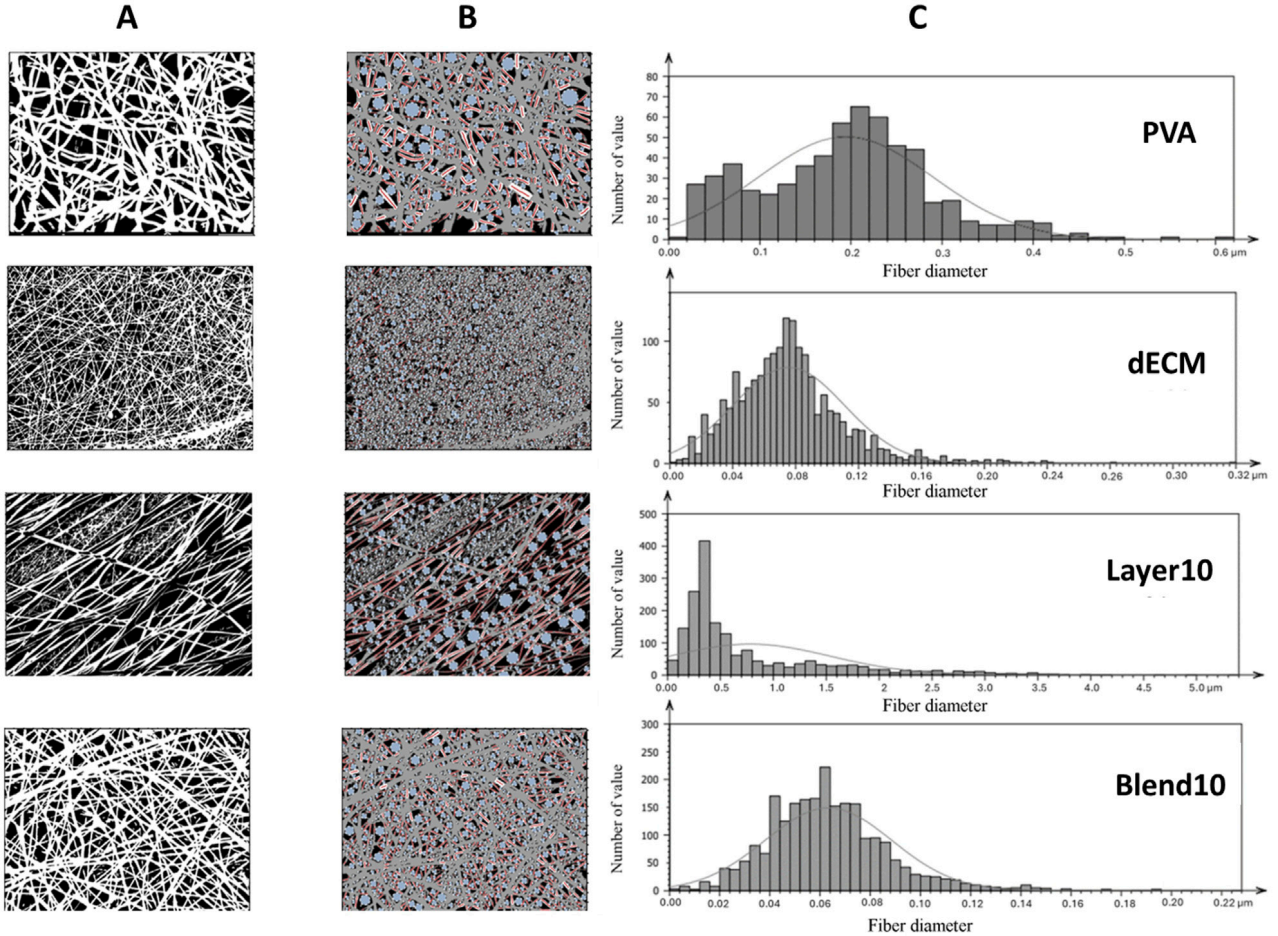
Morphometric evaluation: **(A)** Black and white binarized pictures highlighting the fibers **(B)** inscribed circles in light blue and segmented profiles of the fibers in red **(C)** histograms represent fiber’s average size distribution.

As expected, all matrices showed homogeneous fibers randomly distributed. However, the average fiber diameter and alignment varied depending on the electrospinning setting. Figure 2 reports the binary pictures generated using Mountain8 software after applying the threshold function for morphometric analysis. Figure 2A, and B show binary images of white fibers and black areas with segmented profiles in red and light blue inscribed circles, that were used to evaluate the fibers diameter and the pores, respectively. The histogram illustrates the frequency distribution of the fibers’ diameter (Figure 2C). The average diameter of the fibers in the PVA sample was measured as 0.194 ± 0.096 µm. In contrast, electrospun membranes containing 10% dECM showed a diameter of 0.075 ± 0.035 µm. For composite membranes made of blend of PVA and dECM solution, the fiber size was determined to be 0.063 ± 0.023 µm. The Layer 10 material showed fibers with a significantly larger average diameter of 0.779 ± 0.761 µm, which was statistically different (*p* < 0.05) compared to the other samples (Table 2).

**TABLE 2:** Summary of morphometric parameters analyzed related to fiber size (fiber diameter), porosity (pore diameter), and isotropy. *p < 0.001 respect to relative value of the other conditions.

Samples	Thickness (μm)	Number of fiber	Fiber diameter (µm)	Number of pores	Pore diameter (µm)	Isotropy
PVA	145 ± 65	333 ± 120	0.19 ± 0.10	300 ± 167	0.34 ± 0.22	72.14% ± 0.21
10% dECM	152 ± 67	994 ± 545	0.08 ± 0.04	1265 ± 834	0.18 ± 0.08	66.73% ± 4.05
Layer 10	142 ± 39	1138 ± 716	0.78 ± 0.76 *	897 ± 666	2.34 ± 1.75 *	16.16% ± 3.32 *
Blend 10	122 ± 20	409 ± 289	0.06 ± 0.03	402 ± 376	0.29 ± 0.17	66.02% ± 18.74

Samples obtained through the layering method showed a statistically relevant increase in fibers and pore size and a more aligned fiber orientation compared with all the other samples with a p value < 0.001.

### 3.2 Mechanical properties

**FIGURE 3:**
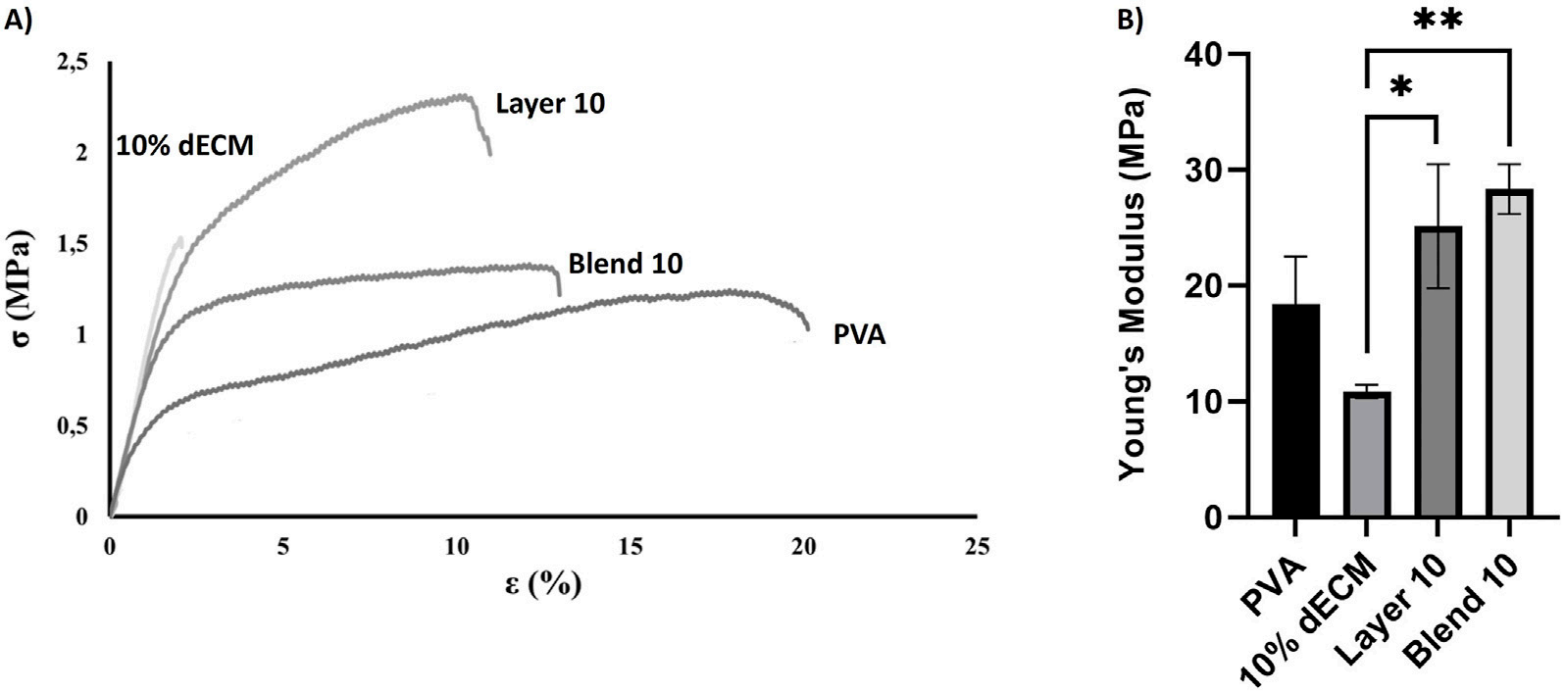
Uniaxial tensile mechanical test: **(A)** Stress-strain curve rapersenting the fracture point and the elongation persentage at breaks **(B)** Young’s modulus measured for different samples. Results are represented as mean ± SD. *p < 0.05 **p < 0.01. n = 3.

The mechanical properties of the electrospun fibers were assessed through uniaxial tensile test, and stress-strain curves were used to estimate Young’s modulus, ultimate tensile strength, and elongation at break (Figure 3). Young’s modulus was derived from the initial linear region of the stress–strain curve.

Elongation at break varied significantly among the samples. Electrospun PVA fibers exhibited the highest deformability before rupture (16.80% ± 1.3%), while fibers containing 10% dECM showed the lowest strain at failure (1.20% ± 0.6%), indicating a more brittle behavior. Layer 10 and Blend 10 displayed intermediate values of elongation at break, measuring 8.9% ± 1.3% and 10.1% ± 3.6%, respectively. Statistical analysis confirmed that Blend 10 and Layer 10 were significantly different from both PVA and 10% dECM (Figure 3A).

Young’s modulus analysis revealed further differences among the groups (Figure 3B). Electrospun PVA fibers showed a modulus of 18.40 ± 4.12 MPa, while the incorporation of 10% dECM reduced stiffness to 10.87 ± 0.59 MPa. In contrast, Layer 10 and Blend 10 exhibited enhanced stiffness, with moduli of 25.12 ± 5.35 MPa and 28.34 ± 2.15 MPa, respectively. These values indicate superior mechanical performance compared to both PVA and 10% dECM.

The stress–strain curves further demonstrate that, as the applied load increases, strain increases proportionally until rupture occurs. At the point of failure, stress drops to zero, and the corresponding strain indicates the fibers extensibility.

Stress–strain curves (Figure 3A) confirm the mechanical trends observed in Young’s modulus and elongation at break. PVA showed the highest extensibility but the lowest strength, fracturing at around 20% strain. In contrast, 10% dECM fractured early, reflecting brittleness. Blend 10 and Layer 10 exhibited improved mechanical strength, with Layer 10 sustaining the highest stress before rupture. These results demonstrate that ECM incorporation and layering strategies enhance stiffness and strength, while reducing ductility.

### 3.3 Contact angle

**FIGURE 4:**
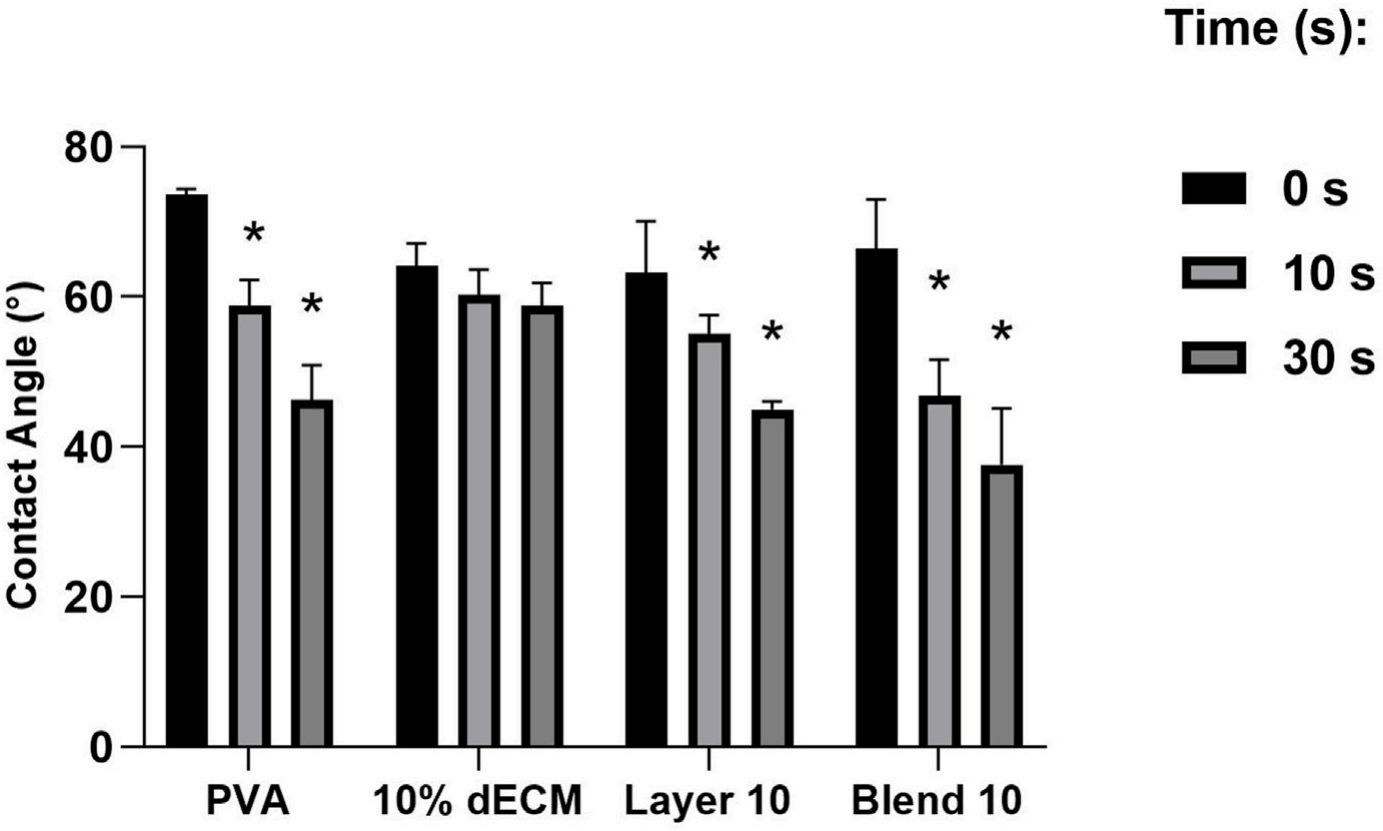
Contact angle of a drop of water on electrospun fibers at (0 s, 10 s, 30 s). Results are expressed as mean ± SD. *p < 0.05. n = 3.

The contact angles of the samples were found to directly correlate with the hydrophilicity of the materials. Notably, the electrospun fibers of PVA, Layer and Blend showed a similar trend with greater degree of hydrophilicity both at 10 and 30 s compared to the dECM (Figure 4). The dECM sample maintained a stable contact angle of 60° throughout the 30 s observation period before the absorption. No significant differences were observed among Layer 10 and Blend 10.

### 3.4 Hemocompatibility analysis

**FIGURE 5:**
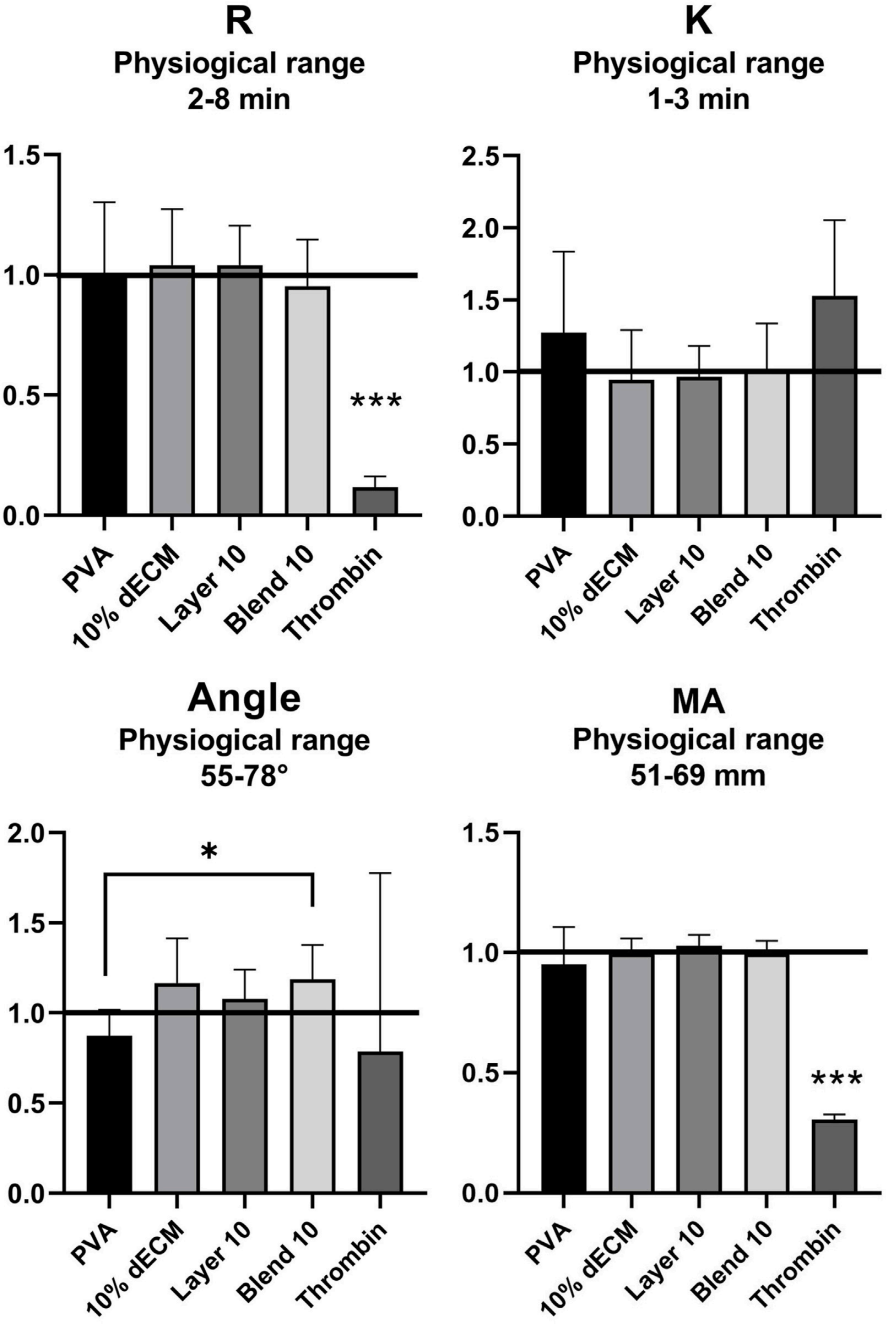
TEG analysis of reaction time (R), clotting time (K), rate of fibrin formation (Angle) and clotting strength (MA) with the relative physiological ranges. The values are normalized respect to the whole blood (black line: R = 4.68, K = 1.65, Angle = 64.92. MA = 65.72). Results are represented as mean ± SD. ***p < 0.001, *p < 0.05. n = 8.

Thromboelastography (TEG) analysis was performed to assess the coagulation profile of whole blood after contact with electrospun membranes. All the untreated blood samples showed values that were within the physiological ranges specified by the manufacturer. After the activation of the blood, no alterations in TEG parameters were observed. Figure 5 shows the results normalized versus untreated blood (black line) related to the main TEG parameters: timing to initiate clotting (R–normal range: 2–8 min), timing of clot formation (K - normal range: 1–3 min), fibrin formation rate (Angle - normal range: 55°–78°), and maximum clot strength (MA - normal range 51–69 mm).

Statistical analysis showed significant differences (*p* < 0.001) for R and MA values of thrombin compared with all the electrospun samples. Furthermore, a statistically significant decrease (*p* < 0.05) was observed in the Angle value between the PVA sample and Blend 10. The thrombin positive control showed statistical differences in terms of clot initiation but showed reduce streght of the clot obtained. The hybrid fibers obtained in the blend strategy are able to increasd, that means that strenght to speed fibrin formation, no other statistically differences have been observed.

**FIGURE 6:**
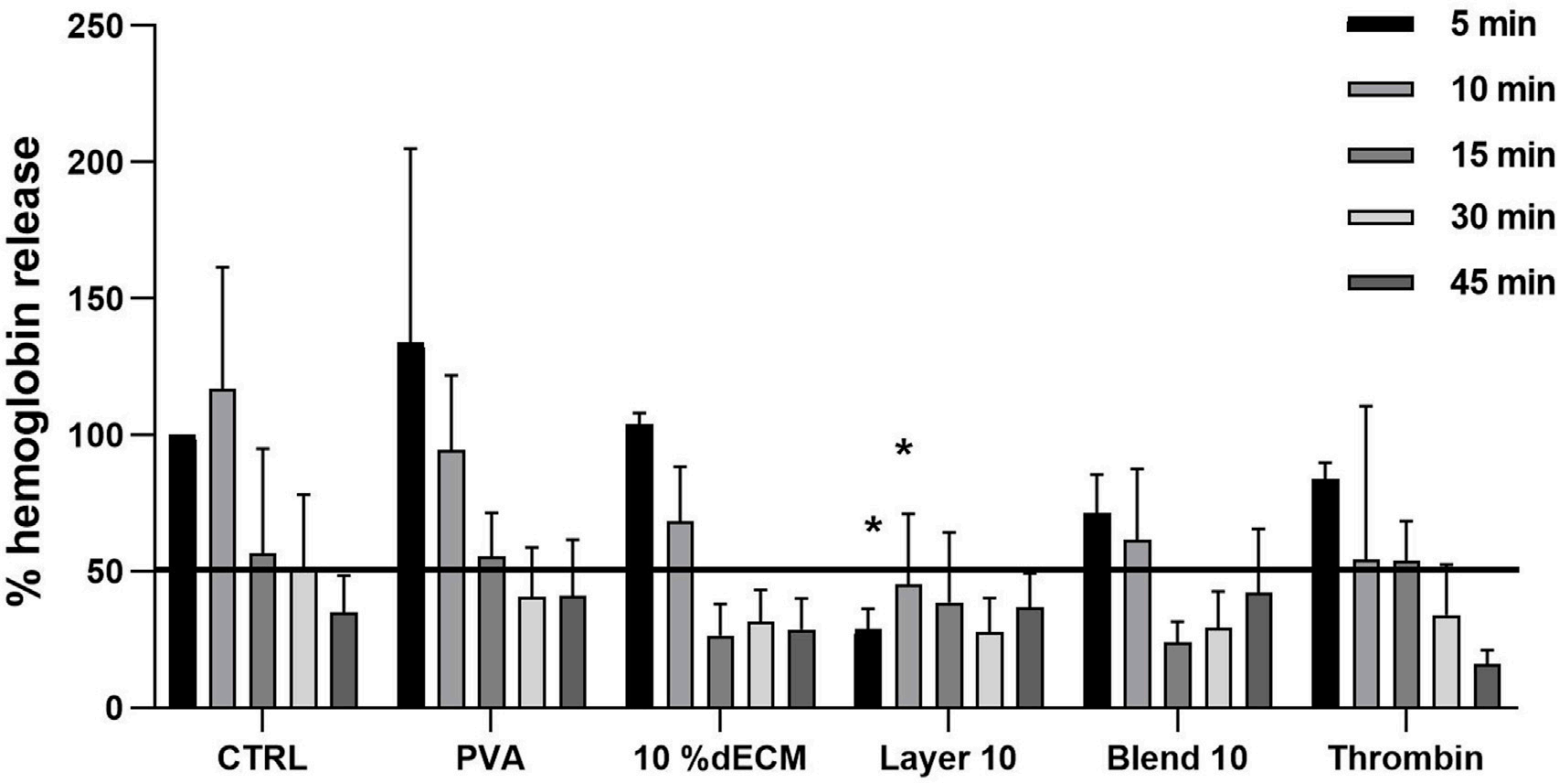
Relative percentage of hemoglobin released from clots after 5, 10, 15, 30, 45 min. Results are expressed as mean ± SD and the statistic was performed respect to the whole blood at the same time point. The black line represents 50% of absorbance, considered as a cut-off for the stabilized clot. n = 5.

To better describe blood interaction with the patches, an *in vitro* coagulation test was performed. Hemoglobin release was normalized based on the whole blood without patches at 5 min. Less than 50% of hemoglobin was released within 30 min in all conditions (Figure 6). Results showed that Layer 10 released a lower amount of hemoglobin after 5 min, showing a statistical difference (*p* < 0.05) with respect to the control, thus, indirectly indicating a stable clot-like structure. All the other conditions required a longer contact time to reach this value. In particular, 10% dECM and Blend 10 released less than 50% of hemoglobin after 10 min. PVA and the control showed a slower coagulation process (30 and 45 min respectively) with a similar trend but with higher inter-donor variability at early time points compared to the samples containing dECM. With the formation of a clot, the amount of hemoglobin entrapped by fibrin increases with time in the early stages of the process.

**FIGURE 7:**
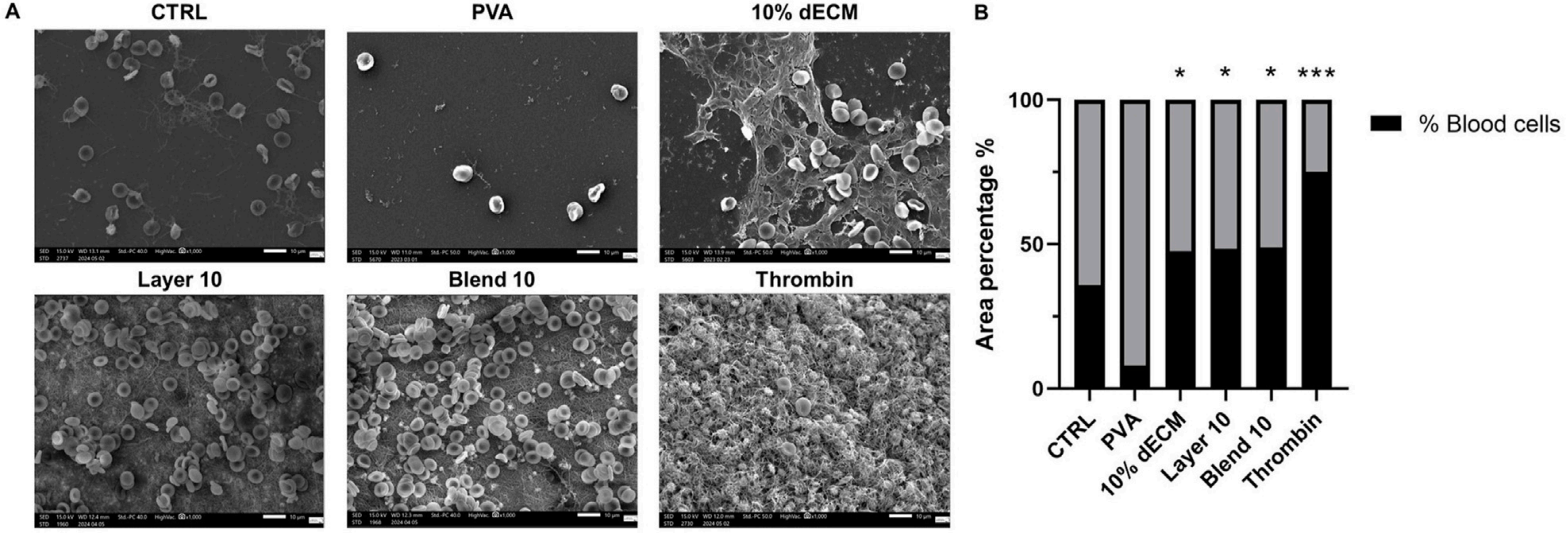
**(A)** SEM images of whole blood clot formation after 30 min at 37°C onto different matrices. Thrombin was used as a positive control. **(B)** Quantification of the area % covered by bood cells, ***p < 0.01, *p < 0.05 compared to PVA sample. n = 3.

To provide additional information on the clot formation and morphology, SEM images of samples placed in contact with whole blood for 30 min were acquired. Figure 7A shows fibrin filaments and red blood cells adhered to the control, indicating minimal clot-biomaterials interactions.

Few adhered red blood cells and minimal fibrin presence was observed on the PVA surface, while dECM samples showed a well-formed blood clot, with fibrin fibers entrapping red blood cells, proportional to the hemoglobin released observed in the *in vitro* clotting test. The area percentage of remaining blood cells in the electrospun containing dECM was comparable in all the samples (around 50%). Morphometrical analysis revealed that all these samples containing dECM showed no statistical difference with respect to the control (35,84% ± 19.68), while the result was statistically increased compared to PVA (*p* < 0.05), where few remaining cells were identified (8.14 ± 4.39 respectively). In the thrombin sample the highest area covered by blood cells was identified (*p* < 0.01). Data are resumed in Figure 7B.

Finally, platelet activation was evaluated using the surface marker CD62P. Elevated CD62P expression is associated with platelet involvement in inflammation, thrombosis, and tissue repair. Thus, CD62P is a widely recognized and reliable early surface marker of platelet activation. The obtained results showed that materials promoted specific platelet activation pathways compared to the control, represented by non-treated PRP. Freshly isolated platelets were used as a control and as a baseline for the relative percentage of platelet activation.

**FIGURE 8:**
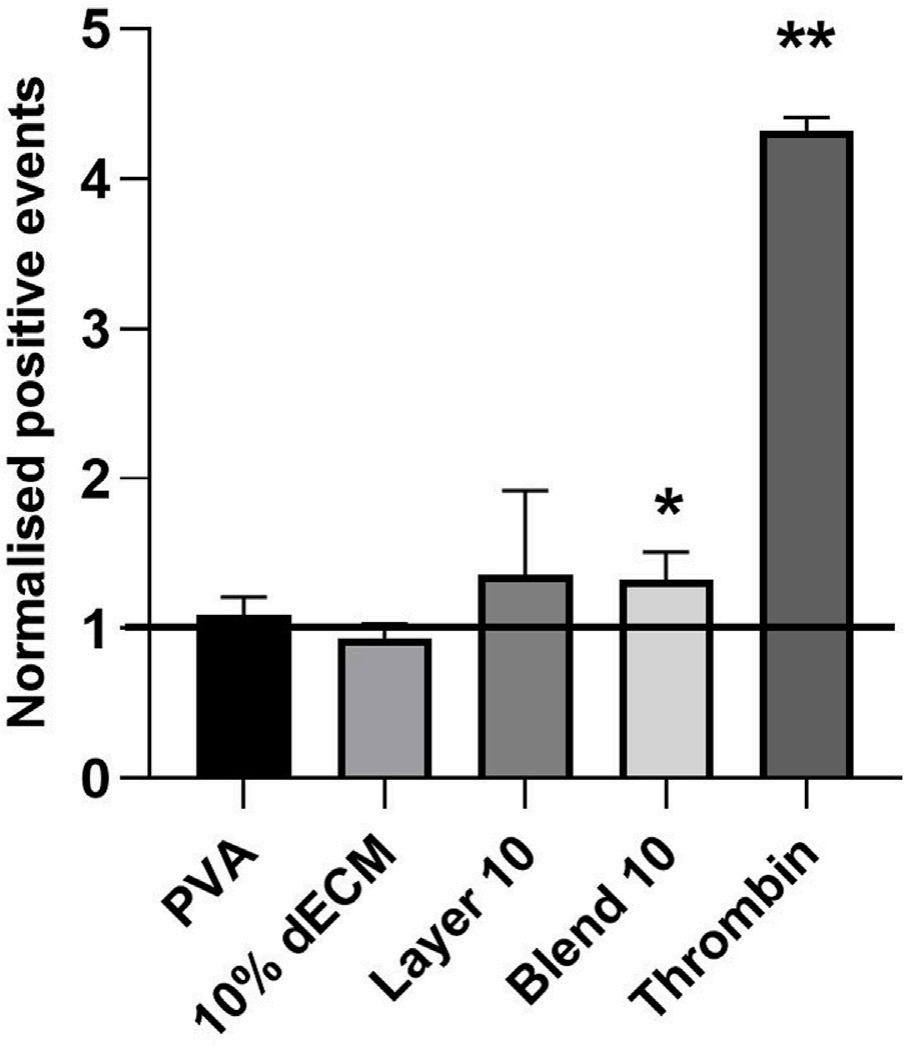
Cd62p expression analyzed by flow cytometry, representing platelet activation in contact with electrospun samples. Results are represented as mean ± SD. **p < 0.01, *p < 0.05. The black line indicates the untreated PRP. n = 5.

Thrombin induced a four times higher activation compared to the control group. Among the tested materials, Blend 10 exhibited a statistically significant difference in activation when compared to the control (Figure 8).

### 3.5 Indirect cytotoxicity and migration assay

**FIGURE 9:**
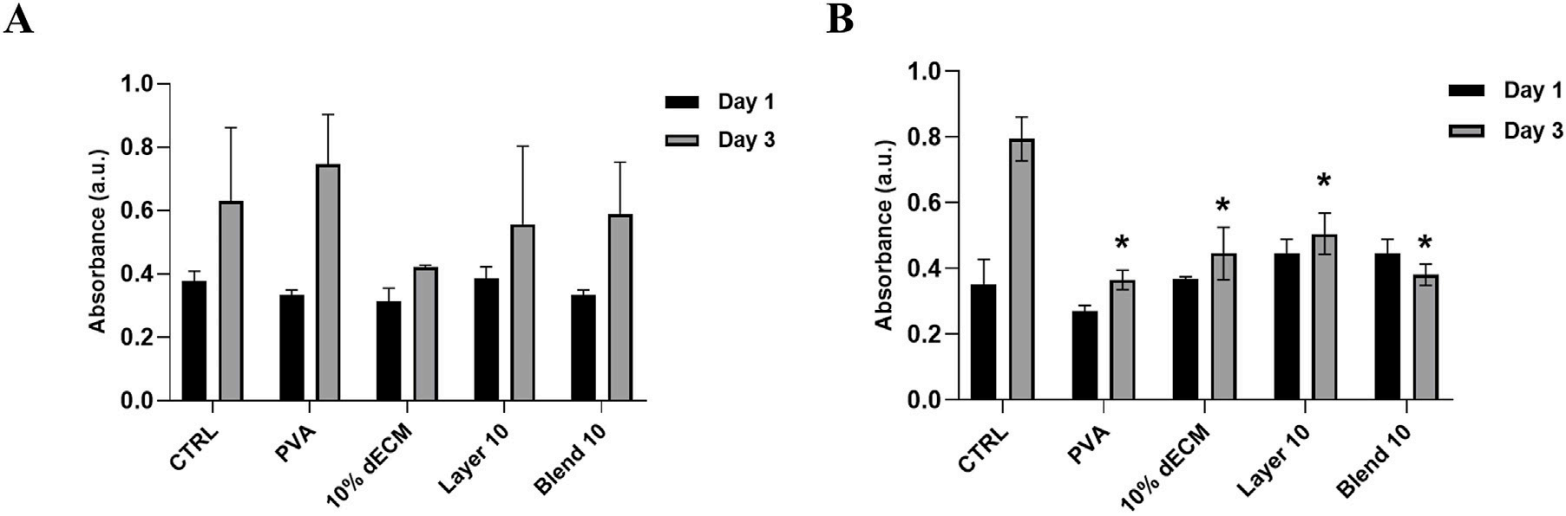
Viability of fibroblast NHDF in conditioned medium for **(A)** 1 day and **(B)** 3 days. Results are represented as means of the absorbance ± SD, *p < 0.05 n = 3.

In order to assess the indirect cytocompatibility referred to the degradation by-products of the electrospun samples, NHDFs were cultured in a medium previously conditioned by incubation with the electrospun materials for 1 and 3 days. As shown in Figure 9, all the conditions with eluates conditioned 1 day showed a trend toward an increase in viability compared to each condition’s day 1 time points, even if this difference was not statistically significant compared to the control. When considering the cells treated with the media conditioned for 3 days, the observed trend was maintained, even if a significantly reduction compared to the control has been observed. (*p* < 0.05).

**FIGURE 10:**
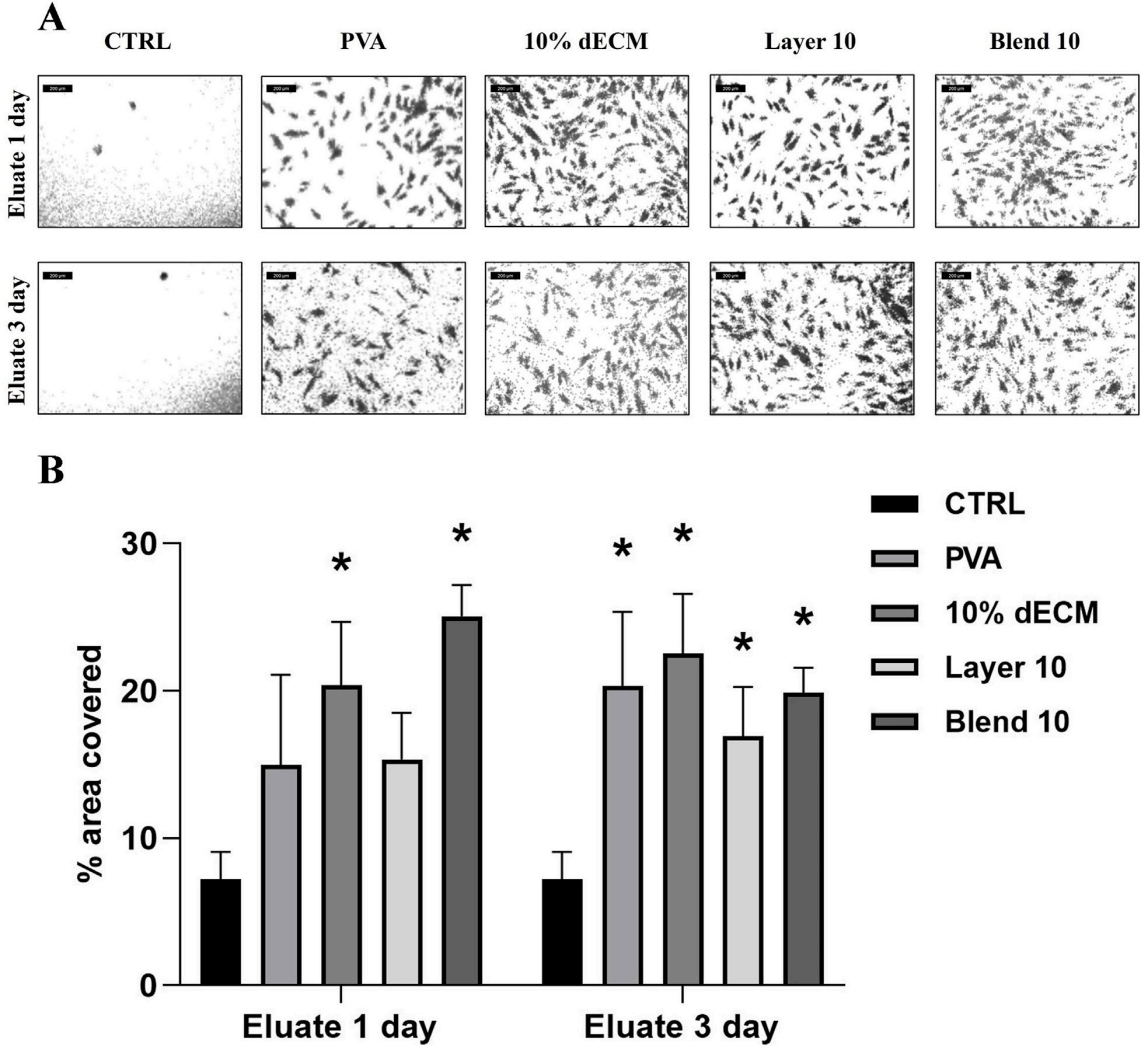
**(A)** images used to measure the area covered by NHDF cells after migration through the transwell membrane after 16 h treated with conditioned medium for 1 or 3 days **(B)** Quantification of the area covered by migrated cells. Results are represented as mean ± SD. *p < 0.05; **p < 0.01. n = 3.

With respect to the chemoattractant properties of the electrospun samples, the migration test showed enhanced cell migration in all the conditions, as demonstrated by a significantly increase in the area covered by migrated cells (Figure 10). In particular, there is a statistically significant difference regarding 10% dECM and the Blend 10 samples on the cells cultured with the conditioned medium for 1 day compared with the control. Furthermore, an increased migration was observed in all the samples treated with the medium conditioned for 3 days.

## 4 Discussion

Nanofibrous materials used as hemostatic patches, when designed to meet specific requirements, offer significant advantages over traditional therapeutic solutions, such as improved bleeding control, enhancement of the healing process, and reduction of preventable complications. In this study, we successfully produced and characterized nanofibrous hemostatic patches composed of dual layers or blends of electrospun materials derived from decellularized bovine pericardium (dECM) and polyvinyl alcohol (PVA). Unlike traditional collagen-based materials, dECM retains different collagen types and other structural components, enhancing the biological properties and providing a biodegradable scaffold that closely mimics native tissues, supporting physiological early responses of platelet adhesion and activation (Bergmeier and Hynes, 2012; Di Francesco et al., 2024).

This study employed two fabrication approaches to develop electrospun hemostatic patches: a dual-layer system with separate PVA and dECM layers and a blended PVA-dECM scaffold. The dual-layer approach was designed to favor the contact of blood with the dECM layer, thus not compromising the native pro-coagulative effects of the extracellular matrix, while the PVA layer improves the processability and strength of the scaffold. In contrast, blended matrices would combine the properties of both materials into a single, streamlined electrospinning process. The study performed by Yao and colleagues demonstrated improved healing efficiency with a bilayer electrospun gelatin-based nanofiber dressing, where a gelatin nanofiber matrix was applied over commercial polyurethane (Yao et al., 2017). Furthermore, Letha and coworkers demonstrated that a double-layered scaffold accelerated wound healing, promoted angiogenesis, and activated coagulation pathways, thereby enhancing the overall regenerative response in treated tissues (Letha et al., 2022). Such bilayer structures highlight the potential of layered electrospun scaffolds in hemostatic applications, where combining bioactivity with structural support can address multiple aspects of wound repair effectively.

Layering and blending could also improve hydrophilicity. Enhanced wettability may promote greater adhesion of blood cells in the short term and potentially accelerate the reabsorption rate of the patch, leading to a more efficient release of bioactive molecules within the nanofibers (Li et al., 2022). Contact angle tests suggest that the formulations containing PVA are more hydrophilic, showing a higher hydrophylicity respect to dECM, which maintains a stable angle throughout the 30 s observation period before water absorption. Even if a stable contact angle suggests that the patches can promote uniform adhesion of blood cells or bioactive molecules over time, materials able to release bioactive components better induce healing during the early stages. In fact, Ma and colleagues worked on electrospun patches composed of PVA and gelatin, highlighting the importance of balancing hydrophilicity and structural stability. The obtained results are in agreement with those of Ma and colleagues, reporting that an optimal contact angle of around 60°, enhances wettability while maintains fiber integrity (Ma et al., 2017). The improved hydrophilicity observed in the PVA-dECM samples compared to dECM alone accelerates degradation over time, aligning well with the intended hemostatic application.

Incorporating PVA additionally enhance mechanical support for the dECM and facilitate the electrospinning process, indicating the blend formulation as an optimal candidate for future investigation for the development of new strategies in the hemostatic field. Furthermore, as suggested by Santos and colleagues, the blending of PVA with biological matrices such as chitosan and cyanobacterial extracellular polymeric substances (EPS) through electrospinning successfully improves mechanical properties and stability of the nanofibers (Santos et al., 2014). In addition, Song and coworkers demonstrated that a blend of collagen, PVA, and hydroxyapatite allows for a consistent electrospinning process, driven by adequate viscosity and electroconductive parameters (Song et al., 2012).

Considering the mechanical stress, Layer 10 and Blend 10 display intermediate extensibility values, with Layer 10 showing slightly lower values than Blend 10 (not significant). This implies that both formulations improve deformation resistance compared to 10% dECM alone, although they do not reach the flexibility of pure PVA. Layer 10 and Blend 10 structures successfully mitigate the weak deformability of dECM, showing improved mechanical resistance compared to 10% dECM, while not reaching the flexibility of pure PVA.

Lu and colleagues developed a PVA and dECM composite bioink for 3D printing, obtaining promising results in term of printability and mechanical properties, achieving good results in meniscus regeneration, thanks to excellent strength and elasticity properties (Lu et al., 2022). Inspired by the advantages of 3D printing applications, a similar PVA and dECM combination for electrospinning was used in this study. This approach was selected to harness the benefits of PVA’s structural support and hydrophilicity alongside the bioactivity of dECM, to address key hemostatic challenges such as rapid clot formation, cellular adhesion, and scaffold resorbability (Gao et al., 2023). The observed comparable mechanical strength results for Layer 10 and Blend 10 suggest that adding PVA to fibers with 10% dECM improves the resistance of the electrospun fibers. This result aligns with findings in similar hybrid scaffolds containing collagen and PVA. Wu and coworkers found that polymer concentration significantly affects the fiber diameter of pure collagen electrospun mats, but all the concentrations tested had poor mechanical properties (Wu et al., 2018). Our results support that hybrid scaffolds incorporating structural and bioactive polymers can achieve improved mechanical integrity while retaining functional benefits, such as supporting bioactivity for hemostatic applications. This approach doesn’t compromise the scaffold’s strength and functional versatility, addressing common limitations in collagen-based matrices.

The fibrillar structures of the ECM provide structural support for cells, enabling them to migrate, adhere, proliferate, and depose matrix components essential for the continuous remodeling of connective tissue (Canciani et al., 2021). In this study, the electrospun patches aimed to mimic connective tissue’s morphological, physical, and mechanical architecture, thereby promoting healing and regeneration of the tegumentary system for research purposes. The electrospinning method is a pivotal requirement to obtain a scaffold able to mimic the natural structure of the tissues with a high level of reproducibility. SEM analysis confirmed that all samples showed a consistent, randomly oriented fibrous morphology with no defects. Only the layered samples showed a significant difference in fiber and pore size compared to the electrospun PVA and dECM samples produced separately or as a blend. This effect may result from using PVA as a separate template on the metallic collector, which likely acts as an insulator and reduces the electric field’s intensity. Consequently, the decreased field strength led to increased fiber diameter in the dECM layer along with the size of the nozzle that has been increased to 24 G to improve the polymer’s acceleration during the electrospinning process. Additionally, the dual-layer configuration showed greater degree of isotropy, possibly due to a narrower Taylor cone and faster deposition, as suggested by Jeckson and colleagues (Jeckson et al., 2021). The observed variations in contact angles are likely to be influenced by an interplay of several matrix surface characteristics, including fiber diameter, porosity, and surface roughness. Smaller fiber diameters and higher porosity enhance surface area exposure, promoting the capillary action and, consequently, faster fluid uptake. Additionally, surface roughness can modulate wettability by altering how water interacts with the material, contributing to the composites’ overall hydrophilicity (Bhardwaj and Kundu, 2010). These results highlight the potential of dual-layer and blended strategies to tune the structure and performance of electrospun matrices for hemostatic applications, depending on the specific clinical requirements in term of stability, bioactivity, and ease of processability. Gao and coworkers reported that a two-phase hemostatic strategy involving a cardiac patch made from electrospun polylactic acid and gelatin doped with resorbable hemostatic particles demonstrating a increased clotting compared to commercial dressings (Gao et al., 2023). The findings obtained in this study report a similar strategy for nanofiber use, as they can be reabsorbed faster, providing comparable hemostatic effects while ensuring enhanced degradability. A similar approach can be considered in the future to shortening the time of clot formation or increase the clot strength, according to specific patients’ needs.

PVA enhance solution processability, allowing for consistent electrospinning, without interfering with the physiological response. However when it was used alone, a slight reduction in fibrin formation was observed morphologically by SEM and by the TEG angle parameter. Conversely, the presence of the dECM promotes hemostasis and wound healing but may hinder processability, stability over time, and mechanical properties (Park et al., 2010). Balancing these components is essential to optimize both electrospinnability and the biofunctional properties of the scaffold.

As reported in literature by Cai and Weng, biomaterials based on decellularized extracellular matrix are able to participate in hemostatic processes due to their high concentration of extracellular matrix protein, including collagen, fibronectin and laminin (Cai and Weng, 2023).

Thromboelastography (TEG) analysis is a research technique that characterizes the viscosity and elasticity of whole blood *in vitro*. In the current study this approach has been used to properly describe the coagulation processes, the fibrinolysis, and the platelet function, making it a valuable tool for understanding blood behavior in clinical scenarios and during biomaterials development (Peng, 2010; Shankarraman et al., 2012; Yang et al., 2022). Our results confirmed that the sample did not affect the coagulation process, showing hemocompatibility in all tested patches in terms of time, fibrin production, stability, and formation of clots (Pretorius et al., 2017). The angle of the PVA control condition obtained by TEG analysis result slightly decreased, indicating that PVA alone slows down the ratio of fibrin formation, a tendency also observed during the SEM morphological evaluation of whole blood, where a less dense and less interconnected fibrin network was detected compared to the Blend 10. This may be due to the high hydrophilicity of the PVA, followed by an immediate dissolution, that seems to slightly interfere with the hemoglobin entrapped in the coagulum before 10 min and in the total number of red blood cells within the. PVA alone drawbacks have been reverted in both Layer10 and Blend10 samples, that remain in the physiological range of all the TEG parameter and showed better clots-matrices interactions overtime. (Song et al., 2012; Santos et al., 2014). On the other side, dECM shows minor hydrophilicity but seems to have greater adhesive properties for blood cells, keeping the clot firmly adhering onto the surface. Furthermore, the results obtained using TEG analysis were supported by the data obtained from coagulation assays and SEM analysis, as reported also in literature. Pretorius and colleagues, for example, demonstrated a good correlation between the morphological analysis of clot formation, assessed by SEM, and thromboelastographic results (Pretorius et al., 2017). In the present project we correlated the results obtained with *in vitro* coagulation test and morphometrical evaluation of the obtained clots in contact with the different patches. The morphometrical quantitative analysis was associated with the hemoglobin release test, enabling the correlation between red blood cells and hemoglobin release. This approach uses a SEM-based method to assess the remaining blood cells after the *in vitro* coagulation, providing insight into the thrombogenic potential. Additionally, measurements on the SEM images were performed by applying Delesse’s principle (Perles-Barbacaru et al., 2012; Nalezinková et al., 2024).

The *in vitro* coagulation test performed on fresh blood revealed that all the materials consisting of dECM exhibited suitable coagulation properties reducing hemoglobin release. Specifically, Layer 10 significantly enhanced the retention of the hemoglobin in the mesh of the fibers peaking at early time points (5 and 10 min), while both Blend 10% and 10% dECM achieved *in vitro* clot formation after 15 min, a faster result compared to thrombin control, that peaked at 30 min and to untreated blood, that peaked at 45 min. Chu and colleagues (Chu et al., 2021) tested chitosan, gelatin and dECM sponges in a similar *in vitro* experimental design (*in vitro* clotting test and platelets adhesion) showing that *in vitro* evaluation only partially mimics the behavior of the coagulation cascade *in vivo*, although the presence of the dECM in the sponges lead to less than 50% hemoglobin release within 10 min, an observation in line with our results. Other studies further highlight the role of nanofiber architecture in optimizing hemostatic performance. Wu and colleagues investigated a hybrid patch composed of N-alkylated chitosan, carbon nanofibers, and PVA. Their study demonstrated that this nanofiber can enhance hemostasis, reduce blood loss, and provide additional antibacterial activity (Wu et al., 2022). In addition, Cheng and colleagues used SEM images to describe stable clot formation in order to select the best oxidation levels for hemostatic cellulose films. Following a similar methodology, as reported in this study, the highest amount of cell adherence, the shape, morphology, and the presence of fibrin fibers were used to evaluate the clot formation qualitatively, to improve the description of the formed clot (Cheng et al., 2013). The correlation between the hemoglobin release test and the morphometric analysis performed on the same tested sample reinforced our findings, highlighting the potential of the samples and their adhesiveness to blood cells in the presence of dECM.

This tendency was significantly reduced in pristine PVA, reinforcing our suggestion that dECM could promote stronger hemostatic interactions.

These morphometrical results suggest that while PVA contributes to hydrophilicity and mechanical reinforcement it may not actively support the fibrin polymerization process as efficiently as dECM containing patches, a tendency observed in our results also in the TEG and *in vitro* clotting test (Cai and Weng, 2023).

Platelet activation is a complex process induced by several agonists, including adenosine diphosphate (ADP), thrombin, epinephrine, thromboxane A2, collagen, and other compounds. This activation triggers platelet aggregation through fibrinogen binding to glycoprotein IIb/IIIa receptors and upregulates CD62P, a key platelet activation marker (Lewis et al., 2018).

Cytofluorimetric analysis revealed that significant differences in platelet activation were observed in Blend 10 and thrombin samples compared to plastic control, indicating that at body temperature (37°C), platelets tend to adhere to a fibrous matrix, aiding red blood cell capture, thus supporting the hemostatic process during *in vitro* analysis (Shi et al., 2016).

Finally, it is crucial to evaluate not only the hemostatic properties of the matrix but also its potential cytotoxicity or ability to support tissue regeneration. The viability of NHDF cells showed no significant differences among all the samples tested, suggesting that the biomaterials were non-toxic to the cells. A significant increase in viability was found between 1 and 3 days for cells cultured with a conditioned medium for 1 day, which is in line with the trend observed for all the samples. When considering the samples treated with the 3 days-conditioned medium, a significant reduction in viability was observed in all samples at 3 days compared to the control. This phenomenon could represent a beneficial cellular adaptation, due to a potential phenotypic switch, as reported in the literature (Chelmuş-Burlacu, Tang, and Pieptu, 2023, Kessler et al., 2001).

These results were further elucidated by the migration assay, in which an increased trend in cell migration in the presence of the conditioned medium was observed, indicating that these scaffolds exert their influence within a relatively short timeframe of 16 h. These scaffolds may induce the migration of fibroblasts towards the injured site, thereby promoting the progression of the granulation phase in tissue repair.

Furthermore, results confirm the importance of controlling fibrous and porous structures to achieve effective cellular adhesion and coagulation, consistent with findings by Cheng and coworkerset al., who reported that freeze-dried PVA/Chitosan sponges with approximately 50% porosity and an average pore size of 100–300 μm demonstrated superior absorption capacity, mechanical strength, flexibility, and pro-hemostatic effects (Cheng et al., 2013).

Future research for potential therapeutic applications will require *in vivo* wound healing and trauma therapy tests, and scalable manufacture of high-quality nanofibrous scaffolds and regulatory compliance depend heavily on strict quality control and economical methods.

## 5 Conclusion

This study highlights significant progresses in developing electrospun dECM/PVA nanofibrous patches. These nanofibrous membranes showed favorable morphological, mechanical, and biological properties, supporting blood cell adhesion, clot formation, and tissue regeneration.

In particular, the morphometric analysis revealed that the layering technique significantly increased fiber and pore diameters while reducing isotropy, indicating that fabrication method strongly influence the microstructural properties of electrospun membranes.

Overall, the mechanical characterization highlighted that PVA is more elastic but dECM can resists to higher strain. Layer 10 and Blend 10 samples provide a balanced profile between strength and extensibility, suggesting its potential for applications requiring both durability and flexibility. In conclusion, electrospun samples containing PVA showed greater hydrophilicity that will facilitate the reabsorption of the patches, however it slightly reduces the clot streght and elongates the time of fibrin formation when not in combination with dECM. In fact, samples containing dECM support the clotting processes, with increased blood cells retention in the composites meshes. The degration products of the electrospun materials are cytocompatible and promote fibroblast migration, promising features for tissue regeneration reserach.

In conclusion, the study presents a basic research-oriented materials supporting clotting processes. Although blood cells interactions with different surfaces has been well observed and characterized, the role in clot formation it was not significant in all the considered context. At the same time, the process has been optimized and the proposed combination of polymers resulted in a improved matrix that rapreseent a valuable starting point for future research aimed to nanofibers’ functionalization according to the desired application.

## Data Availability

The raw data supporting the conclusions of this article will be made available by the authors, without undue reservation.
